# An entomological survey in the Sudanese Guinean environmental transition zone after indoor residual spraying, Chad

**DOI:** 10.11604/pamj.2021.40.189.27903

**Published:** 2021-11-29

**Authors:** Israël Demba Kodindo, Elise Yangalbé Kalnoné, Adoum Mahamat Oumar, Moundai Tchonfinet, Amen Nakebang Fadel, Brahim Adef Abba, Djédion Belemel, Péka Mallaye, Clément Kerah Hinzoumbe

**Affiliations:** 1 Programme National de Lutte Contre le Paludisme, Ministère de la Santé Publique, N’Djamena, Tchad,; 2 Programme National de Lutte Contre l´Onchocercose, Ministère de la Santé Publique, N’Djamena, Tchad,; 3 Programme National de l´Éradication de ver de Guinée, Ministère de la Santé Publique, N’Djamena, Tchad,; 4 Programme National de Lutte Contre la Trypanosomiase Humaine Africaine, Ministère de la Santé publique, Moundou, Tchad

**Keywords:** Malaria, transmission, indoor residual spraying, Sudanese Guinean zone, Chad

## Abstract

**Introduction:**

malaria is a major public health issue in Africa. In Chad in 2019, with 955,243 confirmed cases and 2,955 deaths, malaria is the main cause of consultations. A longitudinal entomological study was conducted in Moïssala Health District. Its objective was to assess the impact of indoor residual spraying with 80% bendiocarb wettable powder on malaria transmission.

**Methods:**

two areas were defined for the study: Dembo, located in the sprayed area, Moïssala, in the untreated area. Two sampling methods were used: pyrethrum spray catches and human landing catches.

**Results:**

sixteen sessions of human landing catches totalling 32 man-nights were conducted and 160 rooms/site were sprayed. Two anopheles were captured in Dembo and 547 in Moïssala. In Moïssala, An coluzzii, An funestus and An rufipes were captured in the rooms and on human bait. An colluzzii and An funestus were captured in pyrethrum spray catches in Dembo. The anophelian human landing catches density was zero in Dembo while it was 8.38 bites/man/night in outdoor and 10.06 bits/man/night in indoor in Moïssala. Only An coluzziiwas found infected in human landing catches and sporozoite index of was 7.46% (10/134) in outdoor and 7.45% (12/161) in indoor in Moïssala. Malaria transmission was estimated at 0.63 infected bites/man/night in outdoor and 0.75 infected bites/man/night in indoor i.e. 229.95 infected bites/man/year in outdoor and 273.75 infected bites/man/year. In pyrethrum spray catches, An coluzzii and An rufipes were the two species found infected in Moïssala with sporozoite indices of 6.70% (23/343) and 20% (2/10) respectively. However, in Dembo, neither of the two captured mosquitoes was found infected.

**Conclusion:**

the indoor residual spraying campaign in the eastern zone of Moïssala has led to the collapse of vectors´ density and aggressiveness. However, its evaluation over a short period of time is not sufficient to assess the impact of malaria transmission in this constant and endemic malaria zone.

## Introduction

In 2019, 229 million cases and 409,000 deaths of malaria were recorded in 87 malaria-endemic countries. The World Health Organization (WHO) African Region shouldered 94% (215 M) of all cases and two thirds of the global deaths continued to affect children under 5 years in sub-Saharan Africa (WHO, 2020) [1].

However, since its creation, the World Health Organization has always had malaria eradication on its agenda [2]. Efforts have been made to eradicate this scourge in many parts of the world [3]. However, in Africa, eradication is being delayed not only for economic reasons, but also because of insufficient knowledge of how the disease is transmitted in the continent's different ecological areas. In Chad in 2020, with 955,243 confirmed cases and 2,955 deaths, malaria is the main cause of consultations in health facilities i.e. 42% [4]. In addition to the loss of human lives, malaria is costly in terms of public health expenditures [5].

Divided into three geo-climatic zones that determine three epidemiological facies of malaria, Northern Chad is desert with no local transmission [6]. The Sahelian Climate Center corresponds to an area with unstable malaria due to short seasonal transmission. The south with a Sudanian to Sudano-Guinean climate is characterized by stable malaria, with long seasonal transmission [7]. Most of the entomological studies conducted in Chad have concerned the Sudanian and Sahelian zone where malaria transmission is attributed to *An gambiae sl, An funestus, An pharoensis* and *An ziemanni* [8,9]. Yet, to date, no survey has assessed the dynamics of malaria transmission in the transition zone between the Sudanese and Guinean zones. However, the epidemiology of malaria depends largely on the biotope.

Our survey focuses on malaria transmission. It was carried out at the request of the non-governmental organization “Médecins Sans Frontière-France (MSF-France)” to evaluate the effectiveness of the indoor spraying campaign carried out in the eastern zone of the Moïssala Health District. Since 2010, MSF-France has been working in the Moïssala Health District in support of the ministry of public health and the national malaria control program. It has developed preventive (awareness, seasonal malaria chemoprophylaxis) and curative (seasonal malaria hospital management unit) activities.

In an effort to strengthen protection against mosquito bites in addition to long-lasting insecticide-treated nets distributed by the ministry of public health, MSF-France considered using Fendona® (a pyrethroid insecticide) for an indoor residual spraying campaign in the eastern zone of the Moïssala Health District. The recommendations of the national malaria control program following a study carried out in 2015 on mosquito susceptibility in the said health district (data presented in another article) finally led to the use of Ficam® WP (insecticides of the carbamates class ideal for vector control). The objective of this study is to evaluate the impact of bendiocarb indoor residual spray (IRS) on the malaria vector composition, behaviour and *plasmodium* infection rate in Southern Chad.

## Methods

**Figure 1:**
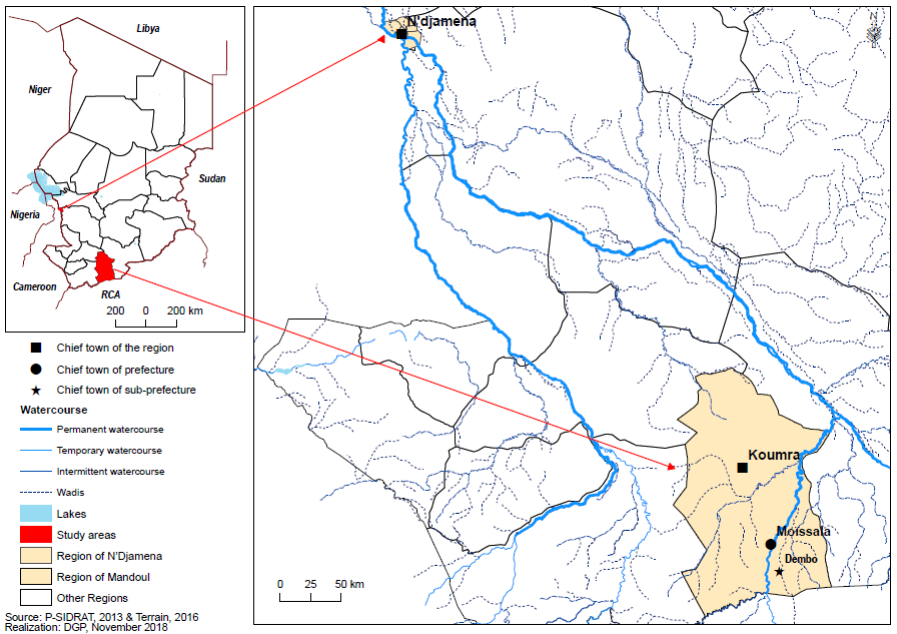
location of study sites

**Study area and population:** to assess the impact of this campaign, two sites were selected. These are Dembo, located in the sprayed area and Moïssala, which was not treated at all, served as a control. Moïssala (8°20'16''N 17°46'05''E) is the capital of the department of Barh Sara, Mandoul Region. It is located 75 km from Koumra, the capital of the region and more than 700 km south of N'Djamena (Figure 1). It has approximately 33,098 inhabitants. Moïssala town is located on the banks of a permanent river, the Barh Sara, a tributary of the Chari river that originates in the Central African Republic. The river can be crossed with a ferry or a canoe. However, during the rainy season and depending on the level of the floods, the crossing with the ferry is not always guaranteed. The alternative is the road for a two-day trip to Dembo.

Dembo is the capital of the Dembo sub-prefecture in the Department of Barh Sara. It is located 25 km east of Moïssala and about 27 km north of the border with the Central African Republic. It has approximately 19,013 inhabitats. The city is bordered by a pond (Goumoud) which is watered in the rainy-season and dried up in the dry season. During rainy season, swamps are formed between Dembo and Moïssala, making traffic extremely difficult. Sometimes, when the rains are very heavy, the road is impassable throughout the season but this difficulty disappears completely towards the end of the dry season.

The two cities have geographical and cultural similarities. The climate is tropical of the Sudano-Guinean type, characterized by the alternation of a long rainy season, from May to November, and a dry season, from December to April. The average annual rainfall exceeds 900 mm/year with a maximum of rainfall in August. The average annual temperatures range from 17°C in December/January to 40°C in March/April. Soils are generally sandy or clayey with a high humus content. In some places, soil impermeability causes swampy areas during the rainy season. The once heavily forested region has undergone intense clearing due to high population pressure.

The forest, which consists of very large trees (fig trees, “caïlcédrats”, tamarind trees, “Karités”, “Nérés” etc.), vines and epiphytic plants, has suffered particularly from human activity, which has largely cleared it to replace it with food crops, but now only remains in the form of highly degraded galleries along watercourses. The main crops are: sorghum, groundnuts, rice, cassava, cotton, beans, sesame and market gardening. The population is composed of Mbaye, Nar, Gor, Ngama, Daye and Peulhs ethnic groups. The activities practiced by the population are: traditional rainfed agriculture, fruit growing, animal husbandry, trade and fishing. Large and small livestock are parked at night in enclosures in the vicinity of houses.

Most of the houses are of traditional, rectangular and circular types, with dry clay or brick walls. Their roofs are made of metal sheet or thatch. The population is supplied with drinking water through traditional wells and boreholes equipped with human-powered pumps. The main diseases of the inhabitants of Moïssala and Dembo are in order of importance from the point of view of the number of cases reported by health facilities: malaria, mainly due to *Plasmodium falciparum* is endemic, malnutrition, measles, meningitis, etc. Entomological data from the region are non-existent. Both cities use the same vector control methods based on the use of long-lasting insecticide-treated nets widely distributed to all households during the 2014 mass campaign. The study took place from 7^th^ to 24^th^ December 2016 in four of the eight districts of the city of Moïssala and in four of the seven districts that make up the city of Dembo (Figure 1).

### Methodology

**Sampling techniques:** mosquito population sampling was carried out by two methods of capture: 1) human landing catches at night (indoor and outdoor); 2) pyrethrum spray catches (Red Can®) during the day.

**Human landing catches:** human landing catches has been studied using the Le Goff *et al*. method [10]. For this method, 3 parameters were determined: 1) the human biting rate (Ma) is the number of vectors biting an individual over a fixed period of time; 2) the sporozoite rate (S) is the number of mosquitoes infected with sporozoïtes divided by the total number of mosquitoes examined; 3) entomological inoculation rate (EIR) is a number of infectious bites per person per unit time EIR = MaS.

The catches were made by volunteers, all young men recruited in each city. These volunteers were previously trained after obtaining their consent. “Men-night” are used both as bait and captors. They were protected from malaria by sulfadoxine-pyrimethamine chemoprophylaxis. Catches were made for four consecutive nights in four neighbourhoods of each site, with one hut per neighbourhood. This corresponds to 16 catch sessions per site, or 32 “men-night”. The neighbourhoods were selected randomly, for each capture point, the consent of the head of the household was obtained for the access of the catchers to their household. For each compound, two catch points are retained. One inside the hut (used as a bedroom) and the other outside. The captures are organized from 6 p.m. to 6 a.m. by two teams of two capturers each. The first team operates from 6 p.m. to midnight and the second from midnight to 6 a.m.

Volunteers are rotated at each capture session (between teams, capture points and houses) to minimize biases due to their individual ability and attractiveness. Each capturer is equipped with a torch, a watch, hemolysis tubes and bags labelled by time slot and bearing the identification of the capture point. In a practical way, each volunteer, seated on a bench/stool/chair, captures with a hemolysis tube the mosquitoes placed on his bare legs up to his knees. The hemolysis tubes containing the mosquitoes are then placed in bags corresponding to catch time slots.

**Pyrethrum spray catches with insecticides:** this method was used for the collection of female endophilic mosquitoes to assess the density of resting mosquitoes in bedrooms. It consists of spraying a pyrethrum solution into bedrooms to collect mosquitoes at rest. The insecticides used are commercial products based on ORO brand pyrethroids (permethrin: 0.25%; tetramethrin: 0.20%; d-ferothrin: 0.01%; piperonyl butoxide: 0.34%). Before each spray, white bed sheets were spread to cover the entire floor of the room and the furniture, then an agent protected by a mask and glasses, after closing the door and windows, sprays the room for about fifteen seconds before leaving. After about ten minutes of waiting, the sheets are carefully removed and the knocked-down-mosquitoes are recovered with the help of pliers in petri dishes bearing indications on the site, the district, the compound and room number. These spraying sessions take place between 6 a.m. and 11 a.m.

**Treatment of captured mosquitoes:** mosquitoes captured by the above methods are identified using a binocular magnifying glass according to the morphological genus and species criteria [11]. Females of anopheles are then counted and classified according to the location, time and method of capture, repletion state of their abdomen (unfed, fed, half-gravid and gravid) and individually deposited in microtubes containing a desiccator (silicagel) for transport and storage. The microtubes are assembled in bags and stored at minus 20°C for further analysis at the laboratory of “Laboratoire du Centre de Recherche Entomologique de Cotonou (Benin)”. The determination of the circumsporozoite antigen of malaria parasites in mosquitoes was done by the Elisa technique [12,13]. Molecular identification of *An gambiae* complex species was performed by polymerase chain reaction short interspersed nuclear element (PCR SINE) 200 according to the protocol of Santolamazza *et al*. [14].

**Statistical analysis:** all data recorded on the survey sheets were entered into Microsoft Excel software and transferred to version 20 of the IBM SPSS statistical analysis software. The human biting rate (Ma) was calculated from the number of bites received per man per night. Letter m represents the number of mosquitoes per man and a, the daily frequency of bites performed by a female anopheles on a man. The sporozoite index corresponds to the presence in females of anopheles of the circumsporozoite antigen established by enzyme-linked immunosorbent assay circumsporozoite protein (ELISA CSP) research. This index is estimated in number of infected female anopheles out of the total number of anopheles analyzed times 100. The entomological inoculation rate (EIR) was calculated from the product of the daily human biting density (ma) and the proportion of females with CSP antigen (s). The daily entomological inoculation rate allows the monthly and annual inoculation rate to be deducted. Anophelian density is the product of endophilic anophelian mosquitoes and the number of rooms sprayed. Fisher test was used to compare the different entomological parameters.

**Ethical considerations:** this work was presented and obtained authorization from the ministry of public health and from the administrative and health authorities of the Mandoul Region. Individual informed consent was obtained from of volunteers who collect mosquitoes and from room owners. The volunteers were trained and protected against malaria chemoprophylaxis based on sulfadoxine pyriethamine.

## Results

**Figure 2:**
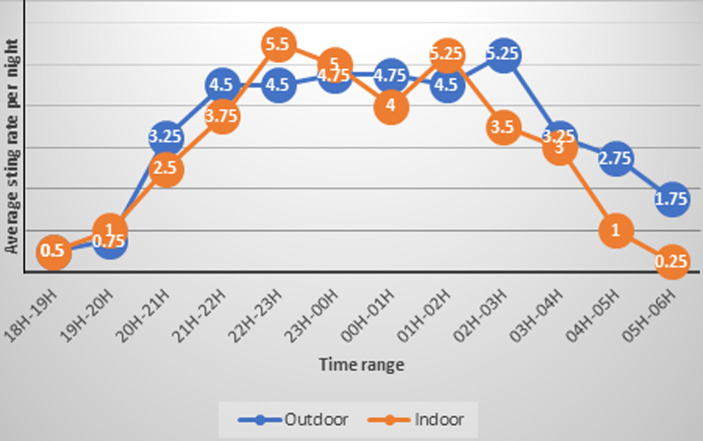
cycle of time human landing indoor and outdoor the rooms in Moïssala

**Human landing catches:** time rhythm and human biting rate: in Moïssala, the host-seeking of mosquitoes starts from 6 p.m. and continues throughout the night both indoor and outdoor. There is a high rate of biting between 10 p.m. and 4 a.m., no matter where you are. Indoor, the bite peak occurs around 3 a.m. while outside, a high rate is reached earlier, between 10 p.m. and 11 p.m., and a second one between midnight and 1 a.m. (Figure 2). During the four successive night captures, 303 *anopheles* were captured in Moïssala while in Dembo, the human biting rate was zero for the same number of capture sessions. The 303 *anopheles* were captured during 16 capture sessions totalling 32 men-night (16 for outdoor collection and 16 for indoor collection). The average human biting anophelian density in Moïssala was 8.38 bites/man/night in outdoor and 10.06 bits/man/night in indoor in Moïssala (Table 1).

**Table 1:** number of mosquitoes collected per site and per collection method

Study sites	Species	PSC (%)	HLC (%)	Total
Moïssala	*An coluzzii*	547 (95.96)	295 (97.36	842
	*An funestus*	13 (2.28)	4 (1.32)	17
	*An rufipes*	10 (1.75)	4 (1.32)	14
	Total	570	303	873
Dembo	*An coluzzii*	1 (50%)	0 (0%)	1
	*An funestus*	1 (50%)	0 (0%)	1
	Total	2	0	2

PSC: pyrethrum spray collections; HLC: human landing catches

*An coluzzii* bites both indoor and outdoor. However, 54.58% (161/295) of the females were caught indoors, indicating the tendency of this species to endophagy. There was significant difference between in outdoor and indoor collection (PË‚0.001). On the other hand, *Anopheles funestus* is exclusively caught outdoors and 75% of *An. Rufipes* are caught outdoors as well. The distribution of the number of anopheles caught on humans is summarized in Table 2 for each species. Sporozoite indices of human landing catches: the sporozoite antigen indices tested in *An funestus* and *An rufipes* were negative. On the other hand, out of a total of 295 *An coluzzii* randomly selected and observed at ELISA for infection, 10 mosquitoes from outdoor collection (10/134) and 12 from indoor collection (12/161) were found to carry plasmodium sporozoites, representing a sporozoite index of 7.46% et 7.45% respectively No significant difference was observed between outdoor and indoor transmission (P=0.99). In Dembo, no test has been performed because mosquito collection was null in this area (Table 3).

**Table 2:** number of species per collection station

Study sites			Collection station		Total
			Outdoor (%)	Indoor (%)	
Moïssala	Species	*An funestus*	4 (100)	0 (0%)	4
		*An coluzzii*	134 (45.42)	161 (54.58)	295
		*An rufipes*	3 (75)	1 (25%)	4
	Total		141	162	303
Dembo	Species	*An funestus*	0	0	0
		*An coluzzii*	0	0	0
	Total		0	0	0

**Table 3:** sporozoite indexes of indoor and outdoor the rooms

Study sites	Especes	O/I	Ma (O/I)	Positive	S (O/I)	EIR (O/I)
Moïssala	*An funestus*	4/0	0.25/0	0/0	0%/0%	0
	*An coluzzii*	134/161	8.38/10.06	10/12	7.46%/7.45%	0.63/0.75
	*An rufipes*	03/1	0.19/0.06	0/0	0%/0%	0
Dembo	*An funestus*	0/0	0/0	0/0	0%/0%	0
	*An coluzzii*	0/0	0/0	0/0	0%/0%	0

O: outdoor; I: indoor; Ma: human biting rate; SI: sporozoites rate

**Figure 3:**
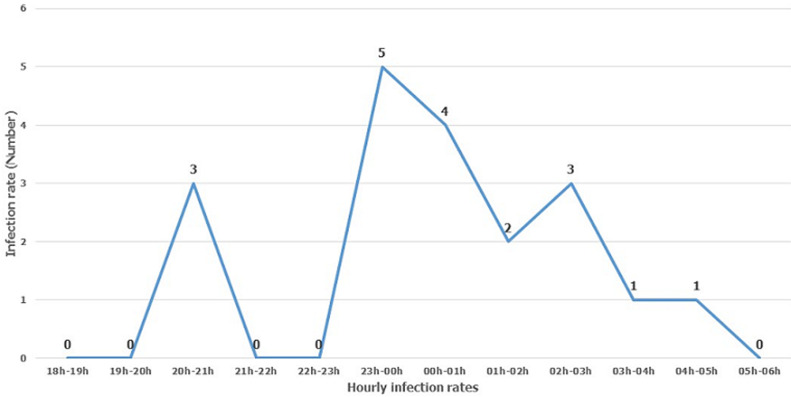
hourly infection rates

The entomological inoculation rate: the average entomological inoculation rate of *An coluzzii* in Moïssala during the study period was 0.63 infected bites per man per night outdoor and 0.75 indoor i.e. 229.75 and 273.75 infected bites per man per year respectively. Time distribution of malaria transmission in Moïssala: hourly host-seeking of Moïssala's mosquitoes starts from 6 p.m. and continues throughout the night. However, the schedules of infected bites are observed from 8 p.m. and persist throughout the rest of the night. However, transmission is null between 6 p.m. and 8 p.m. as well as in the early morning. The high transmission rate occurs during host-seeking peaks, reaching an average of a maximum of five infested bites per person per night during the survey period (Figure 3).

**Pyrethrum spray catches:** density of anopheles per room: during the pyrethrum spray catches, 547 anopheles were captured in Moïssala against 2 in Dembo for a total of 160 sprayed rooms per locality, i.e. an average density of 3.41 anopheles per room in Moïssala against 0.01 in Dembo. *An coluzzii* is by far the most frequent anopheles at rest during the day in houses in Moïssala, while in Dembo the frequency is equal to *An funestus*. In Moïssala, *An funestus* and *An rufipes* are present in small numbers in the houses but in Dembo, the results of the surveys show the absence of *An rufipes* (Table 1).

Sporozoite indices of endophilic fauna: considering the small number of *An funestus* and *An rufipes* captured, all individuals were subjected to the search for plasmodial infection. On the other hand, of the 547 *An coluzzii* collected, 343 were submitted to ELISA for infection detection and 23 were carriers of plasmodium sporozoite antigens, representing a sporozoite index of 6.70%. Two out of 10 *An rufipes* were infected with sporozoites, representing a sporozoite index of 20%. None of the 13 individuals in *An funestus* was found infected. In Dembo, none of the two collected mosquitoes were infected by plasmodium sporozoites (Table 4).

**Table 4:** sporozoite indexes of pyrethrum spray catches

Study sites	Especes	Total	Positive	Sporozoite indexes
Moïssala	An coluzzii	343	23	6.70%
	An rufipes	10	2	20%
	An funestus	13	0	0%
Dembo	An coluzzii	1	0	0%
	An funestus	1	0	0%

## Discussion

Effective malaria control is based on an integrated approach of several strategies [15-17]. For vector control, prevention of malaria transmission through the widespread use of insecticide-treated nets and indoor residual spraying is recommended [18]. First, knowledge of the sensitivity of malaria vector *anopheles* to insecticides and their biting behavior is required before measures can be deployed and post-deployment evaluation of these measures [19,20]. The objective of this study was to assess the impact of the indoor spraying campaign on malaria transmission in the eastern zone of the Moïssala health district after two cycles of bendiocarb spraying. This evaluation was carried out by measuring certain entomological parameters of malaria transmission using methods adapted to the study of vectors.

To better evaluate this campaign, these parameters should have been studied for a year. This would consider the seasonality of transmission and vector density. For example, the results of our study did not allow us to conclude on the degree of effectiveness of internal revenue service (IRS) in the short, medium and long term. Moreover, our results also did not allow us to conclude whether the difference in the specific composition between 2015 and 2016 is related to IRS. However, the results obtained could be used as reliable scientific databases for future vector control planning. The IRS coverage rate was over 85%, which reflects a high level of support from the local population. According to WHO, for IRS to have a positive impact on transmission, a coverage rate of 80% must be achieved. In 2015, five species of anopheles were identified in the Moïssala Health District (*An gambiae sl, An rufipes, An nili, An pharoensis* and *An ziemanni*), while the 2016 results reported only three species: *An gambiae sl, An rufipes* and *An funestus* with a prevalence of *An coluzzii*. These results are comparable to those obtained by Kerah-Hinzoumbé C *et al*. [8] in Goulmoun, Diarra *et al*. [9] in Douguia in Chad and Saotoing *et al*. [21] in Maroua in Cameroon where *An gambiae* predominated with 84.5%, 87.5% and 81.79% respectively during human landing catches and pyrethrum spray catches.

These results could be explained by the similarity of these areas in geoclimatic and ecological terms. The anophelian human landing catches density is 8.38 bites/man/night in outdoor and 10.06 bits/man/night in indoor in Moïssala and zero in Dembo. Moïssala's results are similar to those obtained by Himeidan *et al*. [22] in Sudan, Dossou Yovo *et al*. [23] in Bouaké, Ivory coast, who reported human landing catches density of 8.82 bite/man/year in Um Salala and 8.9 bite/man/night respectively. Dembo's results (0 bites/man/night) would be related to the spray effect. On the other hand, Kerah *et al*. [8] in Goulmoun (Bongor) in Chad reported a significantly higher anophelian human landing catches density of 67 bite/man/night. The density of endophilic mosquitoes is 3.41 anopheles per room in Moïssala and 0.01 anopheles in Dembo. Similar results were reported by Keïta *et al*. [24] along the Niger river in Mali where densities were low in the sprayed huts and higher in the control huts. The difference in densities between Dembo and Moïssala is believed to be linked to the widespread use of insecticide-treated mosquito nets and, for Dembo, coupled with indoor residual spraying. The entomological comparison of the treated area with the untreated area in this study confirms the results that indoor spraying leads to a decrease in vector density and vectors host-seeking [25,26]. This statement, even if it is not shared by some authors [27,28], is confirmed during this survey and seems relevant for malaria prevention even in areas of permanent transmission.

Concerning this parameter, transmission in the control area is about 0.63 infected bites per man per night outdoor and 0.75 indoor. However, it is zero in the treated area. The human biting rate of *Anopheles* indoors (54.58%) is higher than outdoors (45.42%). This study corroborates those of Kerah *et al*. [8], Doannio *et al*. [29] in Ivory coast and Coz and Brengues [30], in Burkina Faso, who observed a rate of human lading catches of 55.8% respectively, significantly higher inside than outside the home.

In Moïssala, anophelian host-seeking behaviour starts from 6 p.m. and continues throughout the night both inside and outside the houses. In both cases, a high rate of bite is observed between 8 p.m. and 5 a.m. However, the maximum is obtained inside between 2 a.m. and 4 a.m. and only decreases significantly after 4 hours. Outside the houses, a high rate was reached earlier, between 10 p.m. to 11 p.m. and the second between midnight to 1 a.m. This intense activity of anopheles between 8 p.m. to 4 a.m. had already been reported previously in some studies [29,31] while others place it after the second half of the night [32]. These results confirm that the host-seeking cycle has not been altered by the mere use of nets in absence of spraying. The biting rate decreases from 2 a.m. outside compared to inside. This decrease in host-seeking would be due to the drop of temperature in December.

Malaria transmission in the Moïssala Health District is due to 2 vectors: *An coluzzii* which has been found infected on human landing catches inside (10.06%) and outside (8.38%) as well as in pyrethrum spray catches (6.70%) and *An rufipes*. Out of 10 *An rufipes* captured in the pyrethrum spray catches and analyzed by ELISA CSP, 2 were positive, i.e. 20%. This result does not make *An rufipes* a secondary vector [11,33] but an excellent one to consider in the transmission of malaria to Moïssala. Our results according to which *An rufipes* is captured at rest in the rooms and on human bait are similar to those observed in Dielmo in Senegal [34] and Dori in Burkina Faso [35]. However, investigations incriminating this species in transmission are rare [36]. Although *An funestus is*, with *An arabiensis*, a major vector of malaria in Chad, this species plays no role in the transmission of malaria to Moïssala.

## Conclusion

The indoor residual spraying campaign in the eastern zone of the Moïssala Health District has led to the collapse of the density and host-seeking behavior of malaria vectors. However, its evaluation over a short period is not sufficient to assess the impact of malaria transmission in this stable and endemic malaria zone. The long-term effectiveness of the indoor residual spraying campaign on malaria vectors and transmission will only be assessed after several years of transmission. In addition, the use of mosquito nets and other control measures should be considered in this assessment.

### What is known about this topic

- Malaria is an endemic vector-borne disease in the Sudano-Guinean zone;
- Insecticide-treated nets are effective against malaria transmission.

### What this study adds

- Bendiocarb used for indoor residual spraying reduces mosquito density and prevents malaria transmission;
- Intra-home spraying can be used in addition to insecticide-treated nets for effective malaria control.
